# Urbanization Increases *Aedes albopictus* Larval Habitats and Accelerates Mosquito Development and Survivorship

**DOI:** 10.1371/journal.pntd.0003301

**Published:** 2014-11-13

**Authors:** Yiji Li, Fatmata Kamara, Guofa Zhou, Santhosh Puthiyakunnon, Chunyuan Li, Yanxia Liu, Yanhe Zhou, Lijie Yao, Guiyun Yan, Xiao-Guang Chen

**Affiliations:** 1 Key Laboratory of Prevention and Control for Emerging Infectious Diseases of Guangdong Higher Institutes, Department of Pathogen Biology, School of Public Health and Tropical Medicine, Southern Medical University, Guangzhou, China; 2 Program in Public Health, University of California Irvine, Irvine, California, United States of America; Mahidol University, Thailand

## Abstract

**Introduction:**

*Aedes albopictus* is a very invasive and aggressive insect vector that causes outbreaks of dengue fever, chikungunya disease, and yellow fever in many countries. Vector ecology and disease epidemiology are strongly affected by environmental changes. Urbanization is a worldwide trend and is one of the most ecologically modifying phenomena. The purpose of this study is to determine how environmental changes due to urbanization affect the ecology of *Aedes albopictus*.

**Methods:**

Aquatic habitats and *Aedes albopictus* larval population surveys were conducted from May to November 2013 in three areas representing rural, suburban, and urban settings in Guangzhou, China. *Ae. albopictus* adults were collected monthly using BG-Sentinel traps. *Ae. albopictus* larva and adult life-table experiments were conducted with 20 replicates in each of the three study areas.

**Results:**

The urban area had the highest and the rural area had the lowest number of aquatic habitats that tested positive for *Ae. albopictus* larvae. Densities in the larval stages varied among the areas, but the urban area had almost two-fold higher densities in pupae and three-fold higher in adult populations compared with the suburban and rural areas. Larvae developed faster and the adult emergence rate was higher in the urban area than in suburban and rural areas. The survival time of adult mosquitoes was also longer in the urban area than it was in suburban and rural areas. Study regions, surface area, water depth, water clearance, surface type, and canopy coverage were important factors associated with the presence of *Ae. albopictus* larvae.

**Conclusions:**

Urbanization substantially increased the density, larval development rate, and adult survival time of *Ae. albopictus*, which in turn potentially increased the vector capacity, and therefore, disease transmissibility. Mosquito ecology and its correlation with dengue virus transmission should be compared in different environmental settings.

## Author Summary

*Aedes albopictus* has expanded its ecological habitat range throughout the world. Although *Ae. albopictus* was previously considered a rural vector, this species has adapted well to suburban and urban environments, and it has become the most important and sometimes the sole vector of dengue virus transmission in urban areas. Dengue is a vector-borne disease that has become a severe global public health problem during the last decade. We explored the effect of ecology in different ecological settings (urban, suburban, and rural) on *Ae. albopictus* larval habitat and mosquito development in Guangzhou, where recently dengue has caused serious public health concerns. The environmental changes caused by urbanization had a significant impact on the ecology of *Ae. albopictus*. Compared with rural and suburban areas, urban areas had more *Ae. albopictus* larval habitats, shorter larval development time, higher adult emergence rate, and longer lifespan. These results imply that urbanization significantly increases the potential for dengue outbreaks. Because urbanization is a global trend resulting from economic development, the elucidation of *Ae. albopictus* adaptation to different environments in China also reveals the potential for this important vector to colonize other parts of the world.

## Introduction

*Aedes albopictus* (Skuse) (Diptera: Culicidae), the Asian tiger mosquito, is an aggressive, strongly anthropophilic, exophagic, and exophilic mosquito. As an important vector of dengue fever, chikungunya disease, and yellow fever, *Ae. albopictus* has emerged as a global public health threat [1]–[3]. *Ae. albopictus* is indigenous to both tropical and temperate regions of Southeast Asia and islands of the western Pacific and Indian Oceans, but it has recently expanded its range to every continent except Antarctica [4], [5]. Unlike wetland mosquito species that oviposit and develop in habitats that are large, predictable, and easy to identify, *Ae. albopictus* is difficult to locate and control because this species utilizes small, different types of habitats including small containers and spare tires [6]–[8].

*Ae. albopictus* originated at the edges of forests and bred in natural habitats (e.g., tree holes, bamboo stumps, and bromeliads) and was previously considered a rural vector [9]. However, this species has adapted well to urban environments with larvae now breeding in artificial containers (e.g., tires, cemetery urns, and water storage containers) and has become the most important and sometimes sole vector in urban areas [8], [10], [11]. *Ae. albopictus* is found almost everywhere, especially in urban areas in southern and southwestern China [12]–[15]. The frequent outbreaks of dengue fever in the cities in southern (mainly Guangdong province) and southeastern coastal (mainly Fujian and Zhejiang provinces) China in the past few decades have caused serious public health concerns [16]–[18]. Although *Aedes albopictus* is described as a minor vector of dengue and possibly chikungunya in the world, it is emerging as a major dengue vector in China and was responsible for most outbreaks of dengue in China [19], [20] and chikungunya in 2010 in Guangdong, China [21].

Similar to other mosquito vectors, *Ae. albopictus* needs aquatic habitats to breed and develop, and therefore, it is sensitive to environmental changes [8], [22], [23]. Destruction of breeding habitats is an important strategy to reduce the *Aedes* mosquito population; eliminating suitable breeding habitats reduces larval development and thus the adult mosquito population. Equally importantly, environmental changes, such as changes in temperature, affect habitat productivity, larval and adult development times, and survival, which in turn directly and indirectly affect disease transmissibility [24]–[32].

Urbanization refers to the increasing population of urban areas. Urbanization predominantly results in the physical growth of urban areas, leading to environmental changes. Urbanization is a global trend that results from economic development. Asian countries including China and India, countries in Southeast Asia, and African countries such as Nigeria are the fastest growing areas in the world, and the unprecedented movement of people into these areas is predicted to intensify in the future [33]. Many problems have emerged as a result of urbanization, including environmental pollution, crowding, and the destruction of natural ecology. The socioeconomic effects of urbanization have been extensively studied by socio-ecologists [34]–[36]; however, the ecological effects and their impact on vector biology and vector-borne infectious disease transmission remain unclear. Most dengue fever outbreaks occur in the urban areas of China, and these outbreaks have become more frequent over the past decade [18], [20], [37]. There is an accelerating trend of urbanization in China; will this process of urbanization accelerate dengue fever outbreaks? Changes in environmental conditions as a result of urbanization may directly and/or indirectly affect the ecology of mosquitoes, e.g., larval habitat availability and suitability, development, and survivorship. Because *Ae. albopictus* has invaded Europe (e.g., Italy and France) and the Americas (e.g., USA), which increases the global vulnerability to dengue fever outbreak, therefore, it is crucial to evaluate its adaptations to urban environments.

We hypothesized that urbanization increases *Ae. albopictus* larval habitats and survivorship and accelerates the development of larvae and adults. This study explored the ecology of *Ae. albopictus* in different settings (urban, suburban, and rural) in the Great Guangzhou area, China. Field surveys of larval habitat availability, larval development and adult mosquito life-table experiments were conducted in semi-natural conditions to test the hypothesis.

## Materials and Methods

### Study areas

The field surveys of larval habitat availability and semi-natural condition larval development and adult mosquito life-table experiments were carried out in Guangzhou, the capital city of Guangdong province, China. Guangzhou is the largest city in southern China, and it is located in the Pearl River Delta, where numerous cities form a Canton-Macao-Hong Kong economic development zone. The annual average temperature in Guangzhou is 21.6°C, and its annual rainfall is approximately 1,980 mm. This climate is ideal for the development and reproduction of *Ae. albopictus*. The city has experienced rapid expansion during the recent regional economic development. Several major dengue fever outbreaks have occurred in this area since 1980, and *Ae. albopictus* is the sole dengue vector [13], [38], [39]. Therefore, Great Guangzhou is an ideal place to study the impacts of urbanization on *Ae. albopictus*.

**Figure 1:**
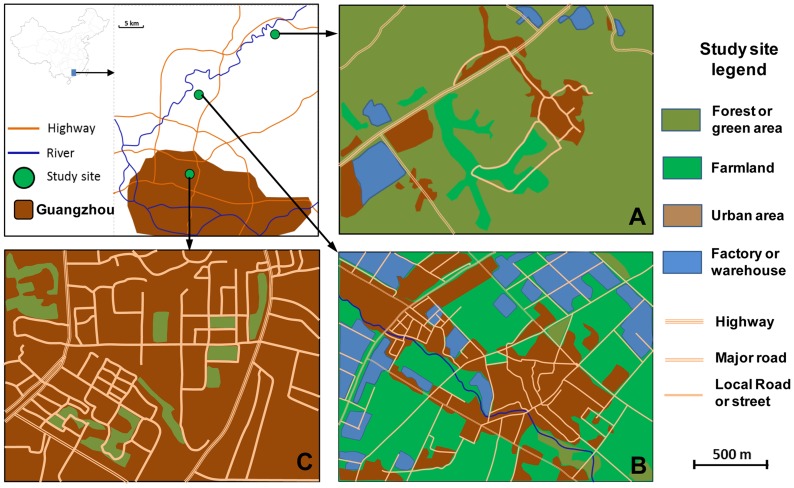
Map of study areas in Guangzhou, Guangdong province, China. The ecology study of *Aedes albopictus* was conducted in three areas. A: Rural, Dengcun in Conghua county, B: Suburban, Liangtian, in Baiyun district, C: Urban, Tonghe in Baiyun district.

The study was conducted in three areas that represented urban, suburban, and rural settings in Guangzhou (Figure 1 and Figure S1). Each study area was approximately 1.8 km^2^. The distance between each area was approximately 24 km. Tonghe (113°19′E, 23°11′N, 31 m above sea level (a.s.l.)) is an urban area with a population density of >3,000 people/km^2^. The land use types are primarily residential and commercial buildings and public services such as schools and hospitals, filled with trees and grasses. Liangtian (113°23′E, 23°21′N, 25 m a.s.l.) is a suburban area with a population density of approximately 1,000 people/km^2^, and land use includes a mixture of residential, manufacturing, and farmland. Dengcun (113°33′E, 23°30′N, 42 m a.s.l.) is a rural area and has a population density of <100 people/km^2^, where land use is primarily agricultural (rice and vegetable planting) and forest.

### Aquatic habitat surveys

Aquatic habitat surveys were conducted in the three areas from May to November 2013. We surveyed all aquatic containers in the study areas monthly with three teams of four trained personnel per team. All properties within the site (i.e., residential, abandoned, commercial, and public services) and alleyways were surveyed, except for parcels whose owners refused access or places that were inaccessible due to physical barriers (e.g., fallen structures). We provided a detailed explanation of the purpose of the study to the residents, and after obtaining consent, we inspected the indoor, outdoor, and surrounding areas for aquatic habitats. The location and the physical characteristics of the habitats were recorded. Their chemical and biological characteristics were sampled, and mosquito larval availability and counts were recorded.

The geographic location of each habitat was located using a hand-held GPS unit (GARMIN Corporation, Taibei, Taiwan). For each habitat identified, the depth and surface area of the water was measured; for small containers (<0.25 L), water was emptied into a separate container to measure the actual volume. Habitats were subjectively characterized by coverage of canopy (direct sunlight, full shade that can be exposed to sunshine, full shade that cannot be exposed to sunshine), habitat type, substrate type (soil, sand, leaf, moss, no substrate), and turbidity (clear = colorless, tinted = in between, polluted = opaque and odoriferous).

### *Ae. albopictus* larval sampling

Immature *Ae. albopictus* samples were classified as young larvae (1–2 instar), old larvae (3–4 instar), or pupae. Immature mosquito abundance was determined using the standard 350 ml dippers. Once dipped, larvae and pupae were collected using a pipette, and individual numbers were counted. Samples were transported to the laboratory, where they were reared until emergence for species identification. All mosquitoes that emerged were pooled by site (Urban, Suburban, and Rural) and species.

### Adult mosquito abundance surveillance

The BG-Sentinel trap with lure (bought from Solbrite Resources Pte Ltd, Singapore; produced by BioGents, Regensburg, Germany) was used for adult surveillance in this study because it is a very efficient tool for capturing adult *Ae. albopictus* [40]–[42]. During the surveys, 12 BG-Sentinel traps were placed in each study area. In each study site, we chose three typical environmental settings for the traps: in the urban area, a residential area, public park, and commercial district; in the suburban area, a residential area, factory, and garden; and in the rural area, a residential area, farmland, and forest. The distance between two traps was at least 50 meters. Traps were placed in the same location for three consecutive days during the first week of each month; they were shifted to different locations for another three days during the third week of each month. The adult population was monitored continuously from July to November 2013.

Trapped mosquitoes were collected every 24 hours, transported to the laboratory, and frozen for species identification. Frozen mosquitoes were placed on a piece of white filter paper in a petri dish on a chill table, and the species was identified morphologically using taxonomic keys [43]. Blood-fed females were identified visually by their dilated red abdomens, and they were stored at −80°C for further analysis.

### *Aedes albopictus* life table experiments

#### Larvae life tables

During our preliminary habitat investigation, we found that rich habitats were available in flowerpots and plastic buckets, plastic buckets and disposal containers, and clay pottery and plastic buckets in urban, suburban, and rural areas, respectively. The water and substrate from these habitats were collected for larval rearing and further life table analysis. *Ae. albopictus* eggs were collected in 2013 from each of the three study areas and reared using microcosms under semi-natural conditions in the respective study sites. Twenty newly hatched (12-h-old) *Ae. albopictus* larvae were placed in plastic bowls (15 cm diameter and 5 cm deep). Each bowl contained 20 g of soil and foliage and 200 ml of water, which were collected locally from the natural breeding containers. Larval life table experiments were replicated 20 times at each site. To determine the impact of food sources on the development of immature mosquitoes, we used tap water supplemented with yeast as a control group in each area; the control was replicated 10 times at each site. All microcosms were screened with white, insect-proof, nylon netting to prevent colonization by other mosquitoes and predators, and they were placed near natural habitats where the canopy cover was >80%. The number of larvae and pupa were counted and the larval stages identified daily. Emerged adult mosquitoes were counted daily, and their sexes were determined. The experiments began in October and were conducted simultaneously in all study sites.

### Adult mosquito life tables

Mosquitoes used for adult life-table experiments were all F0 individuals who originated from different habitats in the study areas. Newly emerged (<24-h-old) adults were transferred to a 30×30×30 cm microcosm covered with nylon netting; 20 females and males each were placed in each microcosm. We used the thumbtack to fix the twine on the ceiling, and cages were lifted 1.0 m above the ground. The twine was smeared with grease to prevent the reach of ants and other insects which may cause interference with the experiment. Cotton wool soaked in 10% sucrose solution was supplied to the mosquitoes daily. Dead mosquitoes were recorded and removed from the cage daily. There was no other mosquito coils or spray near the cages during the experimental period. The experiments were performed in July–August 2013 and repeated in October–November 2013. There were 15 replicates in each site during each season.

### Climatic variability monitoring

Air temperature, humidity, and water temperature were measured using the HOBO data loggers. Data were offloaded using a Hobo Shuttle Data Transporter (Shuttle, Onset Computer Corporation, Bourne, MA) and then downloaded to the computer using BoxCar Pro 4.0 software (Onset Computer Corporation). The monthly rainfall amount in each area was obtained from local meteorological stations (Figure S2).

### Data analysis

The daily average, minimum, and maximum temperatures and relative humidity were calculated from the hourly records. ANOVA post hoc Tukey's honestly significant difference (HSD) tests were used to determine the statistical significance of differences in mean temperature and relative humidity in different areas for each season. Water temperature in larval habitats was analyzed in the same manner. Mosquito larval density was standardized as the number of larvae per liter of water. Differences in immature mosquito density among different areas were tested using the Tukey's HSD test after logarithmic transformation of larval densities. Differences in adult mosquito density among different areas were tested using one-way analysis of variance (ANOVA) with repeated measures after square root transformation of raw data. Survival rates of *Ae. albopictus* larvae were calculated as the proportion of first-instar larvae that survived to emergence of adult. Mean larval development time was defined as the average duration from first-instar larvae to emergence of adult, and was computed separately for each sex. Kaplan-Meier survival analysis was used to determine the effect of different environmental conditions on adult mosquito daily survivorship. Stepwise logistic regression was used to identify the factors significantly influencing the occurrence of immature *Ae. albopictus* in aquatic habitats. We used the χ^2^-test to determine the significance of differences in stage-specific survival rates of immature mosquito from different places, and Tukey's HSD tests of ANOVA post hoc were used to determine the statistical significance of differences in stage-specific development times. Statistical analysis was performed using JMP statistical software (JMP 9.0, SAS Institute Inc., USA).

### Ethics statement

All entomological surveys and collections conducted on private lands or in private residential areas were done with the owners'/residents' permission, consent and presence. These studies did not involve endangered or protected species.

## Results

### Ecological characterization of the *Aedes albopictus* habitat

During our survey period, we found 2639, 2523, and 1760 aquatic habitats in urban, suburban, and rural areas, respectively. χ^2^-test indicated that habitat *Ae. albopictus* positive rate varied significantly (*P*<0.0001) among the three areas, urban area had the highest positive rate (44.0%), then suburban area (37.7%), and rural area had the lowest rate (31.5%). A wide variety of container types were present in the three study sites (Table 1). The most abundant container types in urban areas were plastic buckets (412), and the least abundant were tarps (6). Flower pots, disposable food tins and gutters were also abundant in urban areas (Table 1). The most abundant container types in suburban areas were disposable food tins (665), and the least abundant were pools (2). Abandoned tires, plastic buckets, and clay pottery were also abundant in the suburban area (Table 1). The most abundant container types in rural areas were clay pots (445) and plastic buckets (324); disposable food tins (276) were also found frequently in this area. Overall, the variety of containers and habitat types was less abundant in rural areas (Table 1).

**Table 1:** Summary of *Aedes albopictus* in different study areas and habitat types.

Habitat type^a^	Urban^b^	Suburban^b^	Rural^b^
Plastic bucket	190/412 (46.1)	124/352 (35.2)	95/324 (29.3)
Plastic basin	40/124 (32.3)	72/200 (36.0)	39/141 (27.7)
Flower pot	267/410 (65.1)	10/32 (31.3)	29/63 (46.0)
Disposable food tin	129/379 (34.0)	215/665 (32.3)	71/276 (25.7)
Gutter	120/334 (35.9)	0/9 (0.0)	1/12 (8.3)
Metal container/bucket	89/232 (38.4)	58/183 (31.7)	36/167 (21.6)
Ditch catch basin	32/203 (15.7)	5/93 (5.4)	2/45 (4.4)
Clay pottery	108/181 (59.7)	150/281 (53.4)	177/445 (39.8)
Abandoned tire	113/168 (67.3)	239/441 (54.2)	16/43 (37.2)
Building tool	34/64 (53.1)	46/142 (32.4)	40/79 (50.6)
Terrarium bottle	25/52 (48.1)	4/36 (11.1)	0/17 (0.0)
Pool	4/48 (8.3)	0/2 (0.0)	0/14 (0.0)
Nature container	2/9 (22.2)	10/30 (33.3)	36/74 (48.6)
Tarp	1/6 (16.7)	12/49 (24.5)	6/39 (15.4)
Other^c^	7/17 (41.2)	6/8 (75.0)	6/21 (28.6)
Total	1161/2639 (44.0)	951/2523 (37.7)	554/1760 (31.5)

Note:

a : Over 120 different types of containers were inspected and summarized into the 15 categories listed above. The container type often reflects the name of the container. However, six of the categories include containers that provided comparable larval habitats as follows: “Flower pot” includes ceramic flower pots, plastic flower pots, and aquatic plants; “Metal container/bucket” includes most metal buckets, a few metal dishes, and holds; “Pool” includes natural and man-made pool; “clay pottery” includes clay cylinders and pots; “Building tool” includes barrels of cement, wheel barrows, cement mixers, and cement holds; “Natural container” includes tree holes, stumps, and puddles.

b : The data represent *Ae. albopictus*-positive habitats, total number of aquatic habitats, and percentage of positive habitats in brackets.

c : Containers categorized as “other” included leather shoes, discarded drawers, and wood containers.

Immature *Ae. albopictus* were most often found in abandoned tires (positive rate 67.3%) and flower pots (65.1%) in urban areas (Table 1, Figure S3). In suburban areas, *Ae. albopictus* larvae were common in abandoned tires (54.2%) and clay pots (53.4%) (Table 1). Whereas in rural areas, *Ae. albopictus* larvae were frequently found in plastic buckets (29.3%) and plastic basins (27.7%) (Table 1).

**Figure 2:**
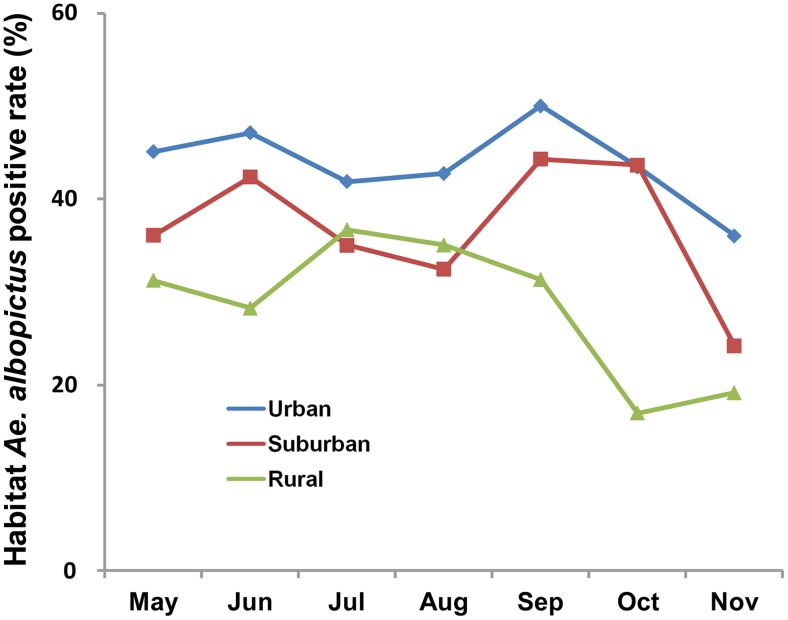
Seasonal shifts in *Ae. albopictus* habitats in different areas.

The number of *Ae. albopictus-*positive habitats varied over time and between different study areas (Figure 2). The urban area had the highest aquatic habitat positive rate in every month except October. Over the seven-month survey period, the aquatic habitat positive rate in urban areas (monthly-mean ± SD 43.8±4.4%) was significantly higher than in rural areas (28.4±7.6%) (Tukey's HSD test, *P*<0.05) but not significantly different from suburban areas (36.9±7.3%).

Densities of immature mosquitoes also varied significantly among study areas (Table 2). The urban area had significantly higher 1–2 instar larvae density than that in the suburban and rural areas, but the difference in 1–2 instar larvae density between suburban and rural areas was statistically insignificant (Tukey HSD test, Table 2). The density of 3–4 instar larvae in urban and suburban areas was significantly higher than that in rural areas, but the difference in 3–4 instar larvae density between urban and suburban areas was statistically insignificant (Tukey HSD test, Table 2). Urban areas also had a significantly higher pupae density than that in suburban and rural areas, but the difference in pupae density between suburban and rural areas was statistically insignificant (Tukey HSD test, Table 2).

**Table 2:** Immature mosquito density at three sites (larvae per liter).

Stage	Urban		Suburban		Rural	
	N	Mean (95% CI)	N	Mean (95% CI)	N	Mean (95% CI)
1–2 instar larvae	2639	7.5 [6.6,8.3] a	2523	6.3 [5.5, 7.1] b	1760	4.4 [3.8,4.9] b
3–4 instar larvae	2639	13.5 [12.3, 14.7] a	2523	12.1 [11.0, 13.1] a	1760	7.8 [6.8, 8.77] b
Pupa	2639	4.2[3.8, 4.5] a	2523	2.1 [1.9, 2.2] b	1760	1.9 [1.7, 2.1] b

Note: Numbers in the same row connected with different letters indicate a significant difference at the 5% level by Tukey HSD test.

### *Ae. albopictus* adult surveillance

**Figure 3:**
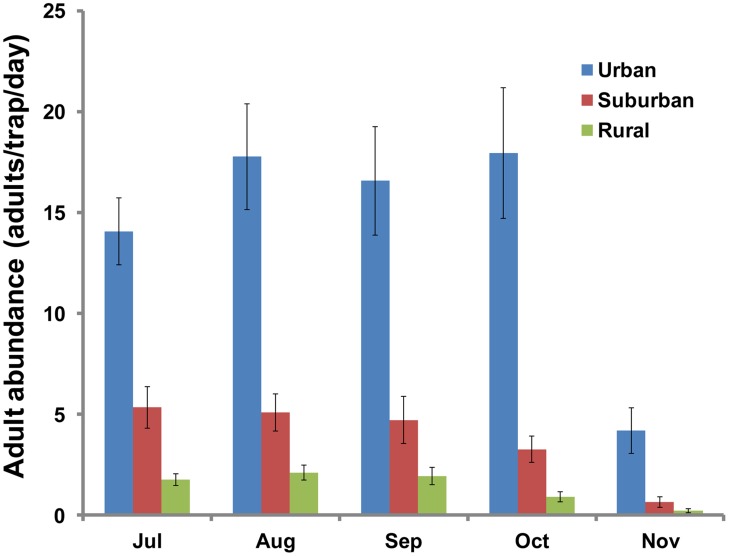
Mean density of adult *Aedes albopictus* (adults/trap/night) in the three study sites from July to November. Square root transformed data were used, and the 95% confidence interval is shown as a bar.

The monthly average density of *Ae. albopictus* adults was significantly higher in urban areas than that in suburban and rural areas, and it was significantly higher in suburban area than that in rural areas (ANOVA with repeated measures, *P*<0.05) (Figure 3). The monthly density of adult *Ae. albopictus* was significantly higher in urban areas than that in the other two sites in all months (Tukey HSD test, *P*<0.05); and it was significantly higher in the suburban area than that in the rural area every month (Tukey HSD test, *P*<0.05) except November (*P*>0.05).

### Life table analysis of immature *Ae. albopictus*

**Figure 4:**
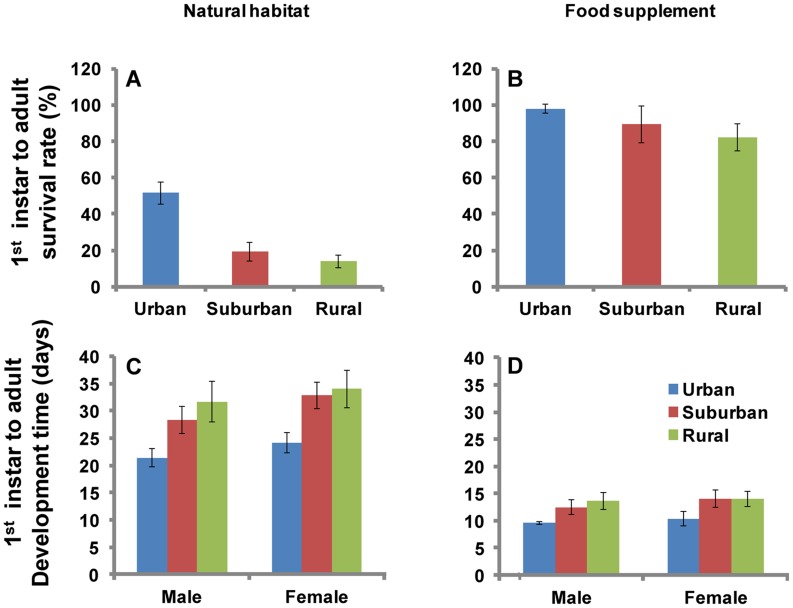
Survival rate and development time of *Aedes albopictus* larvae in urban, suburban and rural areas. A and B: survival rate; A: natural habitat; B: food supplement. C and D: development time; C: natural habitat; D: food supplement. Values are the mean ±95%CI.

*Ae. albopictus* adult emergence rates were significantly different among urban, suburban, and rural areas regardless of in natural habitats or with food supplement groups (χ^2^-test, all *P*<0.001) (Figure 4). In the natural habitat, *Ae. albopictus* adult emergence rates was the highest in the urban area (51.5%), then suburban area (19.3%), with the lowest rate in the rural area (13.9%) (χ^2^-test, all *P*<0.001) (Figure 4A). In the food supplement group, urban areas had the highest adult emergence rate (χ^2^-test, all *P*<0.001), but the difference in adult emergence rates between suburban and rural areas were statistically insignificant (*P*>0.05) (Figure 4B).

For the natural habitat group, larval development time in urban areas was significantly shorter than that in both the suburban and rural areas (male *F* = 19.0, d.f. = 2, 92, *P*<0.001; female *F* = 20.5, d.f. = 2, 98, *P*<0.001) (Figure 4, Table S1). The mean developing time from 1^st^ instar larval to adult were 21.4 and 24.2 days for males and females, respectively, in urban areas, but those values were 28.3 days (male) and 32.8 days (female) in suburban areas and 31.7 days (male) and 34.0 days (female) in rural areas. Habitat water temperature in urban areas (25.8±2.7°C) was significantly higher than that in suburban (20.9±3.4°C) and rural areas (20.5±4.1°C) (*F* = 48.5, d.f. = 2, 174, *P*<0.001). For the food supplemental group, the larval development time from 1^st^ instar larvae to adults in urban areas was significantly shorter than that in suburban and rural areas (male *F* = 20.3, d.f. = 2, 23, *P*<0.001; female *F* = 9.8, d.f. = 2, 23, *P*<0.001) (Figure 4, Table S1). Overall, the larvae to adult development time was >50% shorter in control groups than it was in natural habitat groups in all study sites (Figure 4, Table S1).

**Figure 5:**
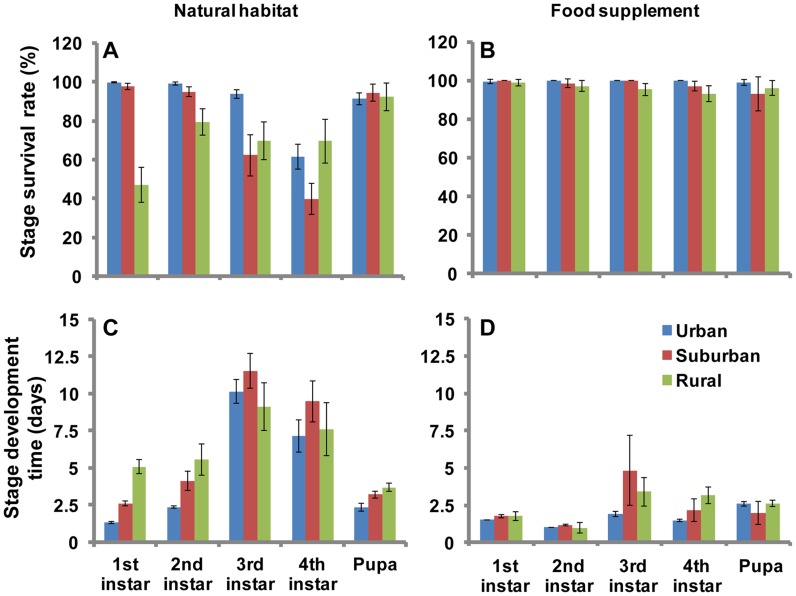
Stage survival rate and stage development time of *Aedes albopictus* larvae in urban, suburban and rural areas. A and B: stage survival rate; A: natural habitat; B: food supplement. C and D: stage development time; C: natural habitat; D: food supplement. Values are the mean ±95%CI.

The larval stage-specific development time were shown in Figure 5. Young larval (1^st^ and 2^nd^ instar) and pupa developed significantly faster in urban areas than that in suburban and rural areas (Tukey HSD test, all *P*<0.001); old larval (3^rd^ and 4^th^ instar) showed similar development time in the three areas (Figure 5). The stage-specific survival rates also varied among the three areas (Figure 5). Overall, young larvae survived significantly better in urban areas than in suburban and rural areas (χ^2^-test, all *P*<0.001). Whereas, survival rates in old larvae and pupae did not show such a difference (all *P*>0.05) (Figure 5).

### Life table analysis of adult *Ae. albopictus*

**Figure 6:**
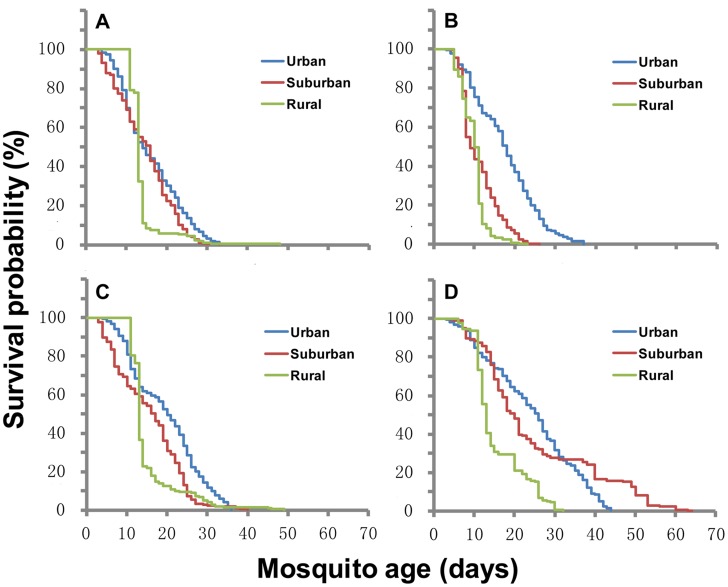
Survivorship curve of adult *Aedes albopictus* in different seasons. The left panel is for August to September, and the right panel is for October and November. The top panel (A and B) is for male, and the bottom panel (C and D) is for female.

From August to September, the life span of female adult mosquito was significantly longer in urban areas than that in suburban and rural areas, but the difference in median survival time between suburban and rural areas was insignificant (Figure 6, Table S2). Adult male mosquito survival time was significantly different among study sites (χ^2^ = 17.4, d.f. = 2, *P*<0.001). Male survival time was longest in the suburban area and shortest in the rural area (Figure 6, Table S2). The average outdoor temperatures in urban (29.4±1.7°C) and suburban areas (29.2±0.9°C) were significantly higher than that in rural areas (28.1±1.8°C) (*F* = 5.4, d.f. = 2, 106, *P* = 0.0016) (Table S2). Relative humidity were significantly different among rural (87.5±9.4%), urban (82.1±11.1%), and suburban areas (75.9±7.6%) (*F* = 10.5, d.f. = 2, 106, *P*<0.001) (Table S2). The mean daily survival rates were similar in all study sites and similar between males and females (Tukey HSD test, all *P*>0.05) (Table S2). Survival curves were similar in females between urban and suburban areas but different from those in rural areas (Figure 6C and 6D).

From October to November, the median survival of adult female mosquitoes in urban and suburban areas was significantly longer than in rural areas but the difference between urban and suburban areas was insignificant (Figure 6, Table S2). The median survival of males was significantly different among the three sites (χ^2^ = 181.1, d.f. = 2, *P*<0.001), with the longest and shortest survival times in urban and suburban areas, respectively (Figure 6, Table S2). The average outdoor temperature in urban areas (24.8±2.6°C) was significantly higher than that in suburban (22.8±3.6°C) and rural areas (21.9±2.4°C), and there was no difference in temperature between suburban and rural areas (Table S2). There was no significant difference in the relative humidity among the three areas. (*F* = 1.9, d.f. = 2, 134, *P* = 0.15). Mean daily survival rates were similar in all study sites but differed between males and females (Figure 6, Table S2). Survival curves were similar in females between rural and suburban areas but very different in those from urban areas, which showed prolonged survivorship (Figure 6D).

### Factors influencing the presence of immature *Aedes albopictus*

Stepwise logistic regression revealed that six factors were significantly associated with the presence of immature mosquitoes in the study sites (Table 3). Habitat *Ae. albopictus* larval presence rate was significantly greater in the urban area than in suburban and rural areas (*OR* = 1.71, *P*<0.001), and the suburban area was significantly higher than the rural area (*OR* = 1.67, *P*<0.001). The presence of *Ae. albopictus* larvae was significantly in negative correlation with habitat water depth (*OR* = 0.03, *P*<0.001); whereas, it showed positive correlation with habitat water surface area (*OR* = 3.95, *P*<0.001). The presence of *Aedes* larvae was significantly greater in clean water than that in tinted or polluted water (*OR* = 1.889, *P*<0.001), and greater in tinted water than polluted water (*OR* = 1.78, *P* = 0.034). Shading (regard less of fully shaded or half-shaded), compare to open area, was positively affecting the presence of *Aedes* larvae (*OR* = 2.29, *P*<0.001). The presence of *Ae. albopictus* larvae was also positively correlated with habitats that have leaves on water surfaces (*OR* = 2.25, *P*<0.001), and with habitats that have soil and moss substrates (*OR* = 1.71, *P*<0.001).

**Table 3:** Factors that were significantly associated with the presence of immature *Aedes albopictus*.

Term	Sub-term	Estimate	Chi-square	P	Odds ratio (95% CI)
Intercept	-	0.80	109.10	<.0001	
Surface area (m^2^)	-	0.14	17.70	<.0001	3.95 [2.11, 7.60]
Water depth (m)	-	−0.04	108.54	<.0001	0.03 [0.01, 0.05]
Study area	Urban vs. Suburban and Rural	0.27	89.94	<.0001	1.71 [1.53, 1.91]
	Suburban vs. Rural	0.25	47.55	<.0001	1.66 [1.44, 1.92]
Water clearance	Clear vs. tinted and polluted	0.32	20.75	<.0001	1.88 [1.44, 2.48]
	Tinted vs. polluted	0.29	4.51	0.0338	1.77 [1.05, 3.03]
Surface type	Leaf vs. no substrate, soil, moss, and sand	0.41	189.44	<.0001	2.25 [2.01, 2.53]
	Soil and moss vs. no substrate and sand	0.27	57.19	<.0001	1.71 [1.49, 1.97]
Canopy cover	Full shade vs. full sun and partial shade	0.41	178.50	<.0001	2.29 [2.02, 2.58]
	Full shade vs. partial shade	0.30	69.75	<.0001	1.81 [1.57, 2.07]

Note: A stepwise logistic regression was used. The presence of immature *Aedes albopictus* was used as the dependent variable, and variables that were insignificant at the 0.05 level are not included in the table.

## Discussion

Outbreaks of dengue fever in China were reported in Hainan province and southern Guangdong province in the 1980s and have been reported in Zhejiang province in 2004, illustrating a 2,000 km expansion from subtropical to temperate areas over 30 years [16]. Among these outbreaks, *Ae. albopictus* was the only vector reported [13], [20], [44]. Although the causes of dengue fever outbreaks are multi-factorial, environmental changes such as urbanization may be one of the leading factors. We found that in urban areas, there are more *Ae. albopictus* habitats. In addition, urban areas promoted faster larval and pupal development, and higher larval-to-adult survival rate compared to rural areas. *Ae. albopictus* mosquito is strongly anthropophilic and has a higher blood-feeding rate in urban areas, where human population density is great, than that in rural areas [29], making it a more susceptible vector in urban areas. Because there is no effective drug therapy or vaccine for dengue fever, vector population control is by far the only effective method for reducing dengue virus transmission. In this context, understanding the vector ecology and biology is essential for developing dengue control strategies. Unfortunately, it is unclear how urbanization impacts the ecology of *Ae. albopictus*, and the lack of this key knowledge hinders disease control efforts.

We found that, in the similar sampling area, the total number of potential habitats and the number of *Aedes*-positive habitats were significantly higher in urban areas than in suburban and rural areas. Urban areas have 10-fold higher human population density and more frequent human activities than do suburban and rural areas, leading to a larger number of artificial containers such as abandoned tires, disposable food tins, and flowerpots, which are all favorable breeding habitats for *Ae. albopictus* [7], [23], [45]. Larger size and higher density of human populations also mean more opportunities for *Ae. albopictus* blood feeding. Previous study found that *Ae. albopictus* has a higher blood-feeding rate in urban areas than in rural areas, most likely due to host availability [29]. Additionally, the existence of stable and abundant artificial containers produced by human activities serve as larval development sites, facilitating large mosquito densities in urban areas [11].

Urbanization shifts mosquito breeding sites from natural habitats to artificial habitats. These artificial habitats are usually small containers such as used tires and disposable containers, and they are often directly exposed to sunlight. Therefore, the water temperature in these habitats is higher than in rural areas. In our study sites, the average water temperature of urban habitats was 5°C higher than in suburban and rural areas. Similarly, vegetation changes and land use changes in urban areas may affect the radiation budget and energy balance of the land surface and thus may modify the microenvironments, e.g., food sources that enhance larval survival. These changes facilitate the development of immature *Ae. albopictus*, i.e., shorten the larval-to-adult development time and enhance the larva survival rate. Our findings are consistent with other studies conducted in different countries and for different mosquito species [24], [30], [46]–[48]. Compared with the food supplemental group, we found that added food sources significantly affect the developmental time and survival rate of immature mosquitoes, which implies that the habitat types in different areas may affect larval development differently due to the difference in availability of nutrients. However, the effects were more pronounced in urban areas than in suburban and rural areas, implying that other factor such as water temperature may play a more important role than food supply in urban areas. These results demonstrated that larvae develop and survive better in urbanized areas, in other words, *Aedes* larvae is better adapted to urban environment.

Similar to a study conducted in the United States [49], the urban area had a higher pupal and larval density than other two areas; thus, the urban habitats had a higher capacity to support larval development. The reason might be that urban areas had less predators, more nutrition from a “dirtier” environment, or even less drift from agricultural insecticides. Pupal productivity is a good indicator of the abundance of adult mosquitoes [50]–[52]. The surveillance of adult mosquitoes in this study supports this conclusion, i.e., urbanization leads to a higher population density of adult *Ae. albopictus*.

Higher mosquito density does not necessarily lead to increased disease transmission if adult mosquitoes have a very short life span. We found that both male and female mosquitoes in urban areas had the longest life spans. This result may be due to environmental factors such as air temperature and humidity. The average temperature in urban areas is higher than in suburban and rural areas. Longer adult life spans may enhance disease transmission, although the exact correlation between vector capacity and adult life span needs to be further explored. In this study we fed the adult mosquito with 10% sugar solution without blood, which might have led to exerted stress on the females during multiple gonotrophic cycles and affected the longevity of the female mosquitoes. We observed that the mortality of adult mosquitoes in rural area changed dramatically around day 15, because the air temperature in rural area showed a 5.7°C increase from day 11 to day 15 compared to the first 11 days. This drastic increase in temperature might have influenced the mortality rate of the adult mosquito.

In our survey, we found that the distribution of immature *Ae. albopictus* was not random. Habitat surface area, canopy coverage, water turbidity, water depth, and substrate type were all important factors influencing habitat selection. These findings confirmed other studies reporting preferences for urban areas [11], [49], shaded containers [49], clean water [53], water with foliage [49], and larger surface area [49], [54]. These results illustrated the complex ecology of *Ae. albopictus*, which makes controlling this mosquito species difficult in light of its recent global expansion.

In conclusion, the results of this study indicated that urbanization has a significant impact on the ecology of *Aedes albopictus*. In the urbanizing and urbanized area, the changed environment became more suitable for the growth and development of *Ae. albopictus*, the condensed population produced more kinds of containers for larval habitats and more blood sources for adult replication. This might be the reason for quick adaptation of *Ae. albopictus* in urban areas. The epidemic of dengue is largely dependent on vector population. Developing countries such as China and other Southeastern Asian countries experiencing rapid urbanization are under sustained risk of dengue outbreaks.

## Supporting Information

Figure S1 **Landscape of study areas in Guangzhou, Guangdong province, China.** A and B: Urban; C and D: Suburban; E and F: Rural.
(TIF)

Figure S2 **Weekly temperature, humidity, and half-month precipitation data in urban, suburban and rural areas in 2013.** A: Temperature; B: Relative humidity; C: Precipitation in one half month. A and B: Values are the mean ± standard error.
(TIF)

Figure S3 **The most abundant** ***Aedes albopictus*** **breeding habitats in the three study sites.** Urban area: (1) aquatic plant, (2) plastic bucket, (3) disposable food tin, (4) gutter, (5) tire; suburban area: (6) tire, (7) disposal food tin, (8) clay pottery, (9) plastic bucket, (10) plastic basin; rural area: (11) clay pottery, (12) plastic bucket, (13) disposal food tin, (14) plastic basin, (15) building tool.
(TIF)

Table S1 **Development of** ***Ae. albopictus*** **larvae.** Note: Values are the mean ± standard deviation. Values in the same column within the same experimental group connected with the same letter indicate a significant difference at the 5% level.
(DOCX)

Table S2 **Life table analysis of** ***Ae. albopictus*** **adults.** Note: Values are the mean ± standard deviation. Values in the same column connected with the same letter indicate a significant difference at the 5% level within the same experimental group.
(DOCX)

File S1 **Original data of the manuscript.** Data used for making the figures and tables for the manuscript.
(RAR)
