# Supplementary figures and images for: Urbanization Increases Aedes albopictus Larval Habitats and Accelerates Mosquito Development and Survivorship
Source: PLoS Negl Trop Dis. 2014 Nov 13;8(11):e3301. doi: 10.1371/journal.pntd.0003301 (PMC4230920; doi:10.1371/journal.pntd.0003301)

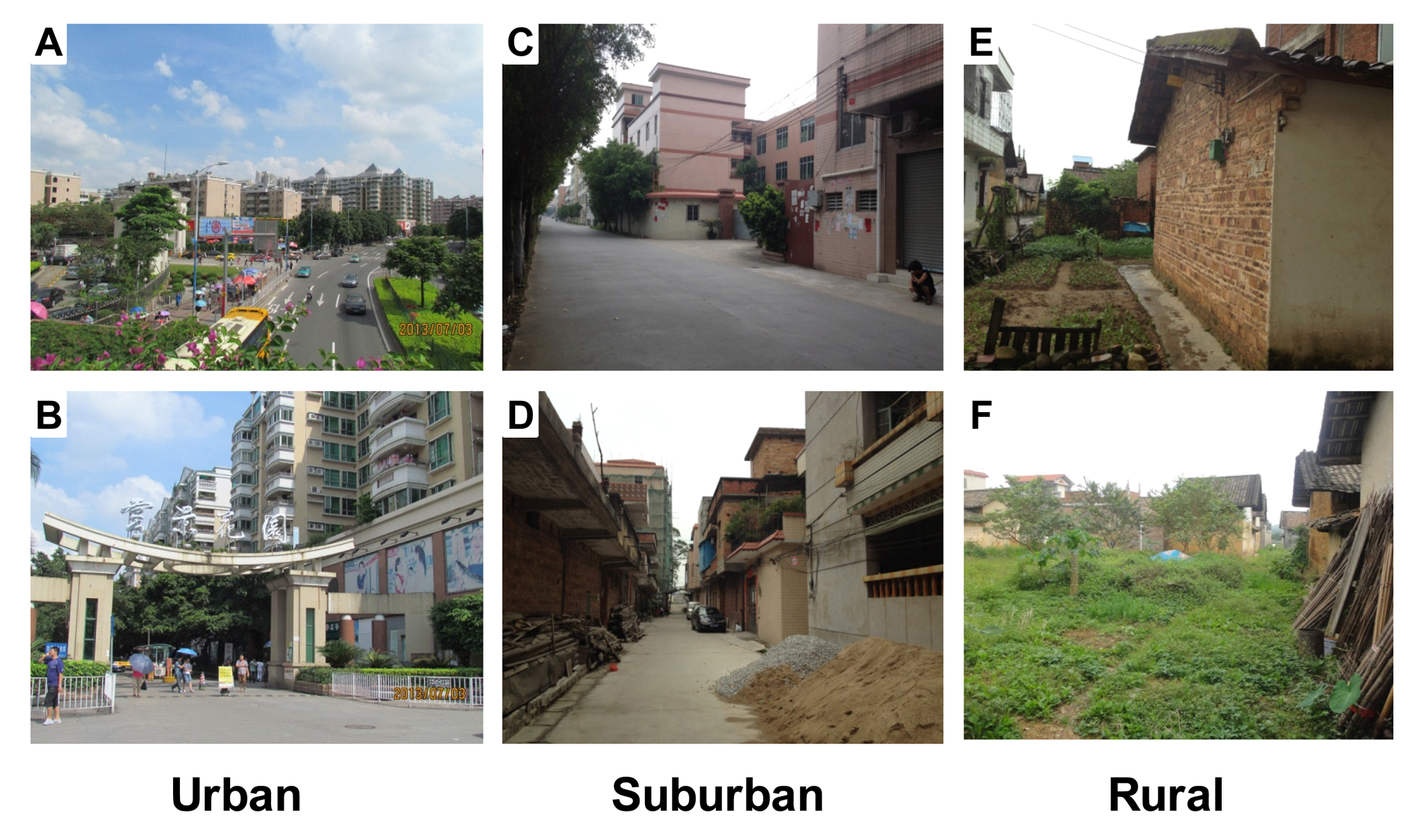

Supplement: Figure S1 — Landscape of study areas in Guangzhou, Guangdong province, China. A and B: Urban; C and D: Suburban; E and F: Rural. (TIF) [file pntd.0003301.s001.tif]

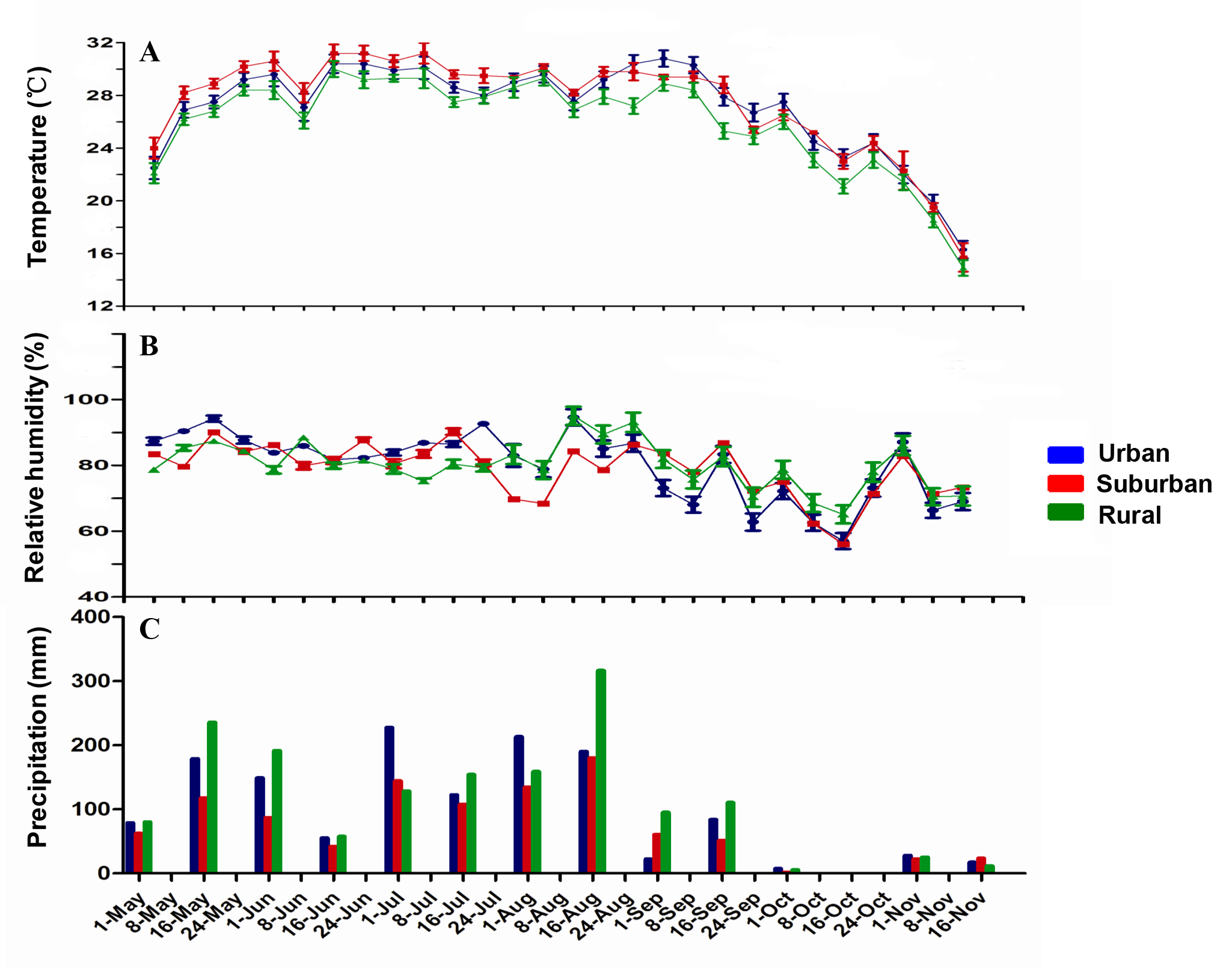

Supplement: Figure S2 — Weekly temperature, humidity, and half-month precipitation data in urban, suburban and rural areas in 2013. A: Temperature; B: Relative humidity; C: Precipitation in one half month. A and B: Values are the mean ± standard error. (TIF) [file pntd.0003301.s002.tif]

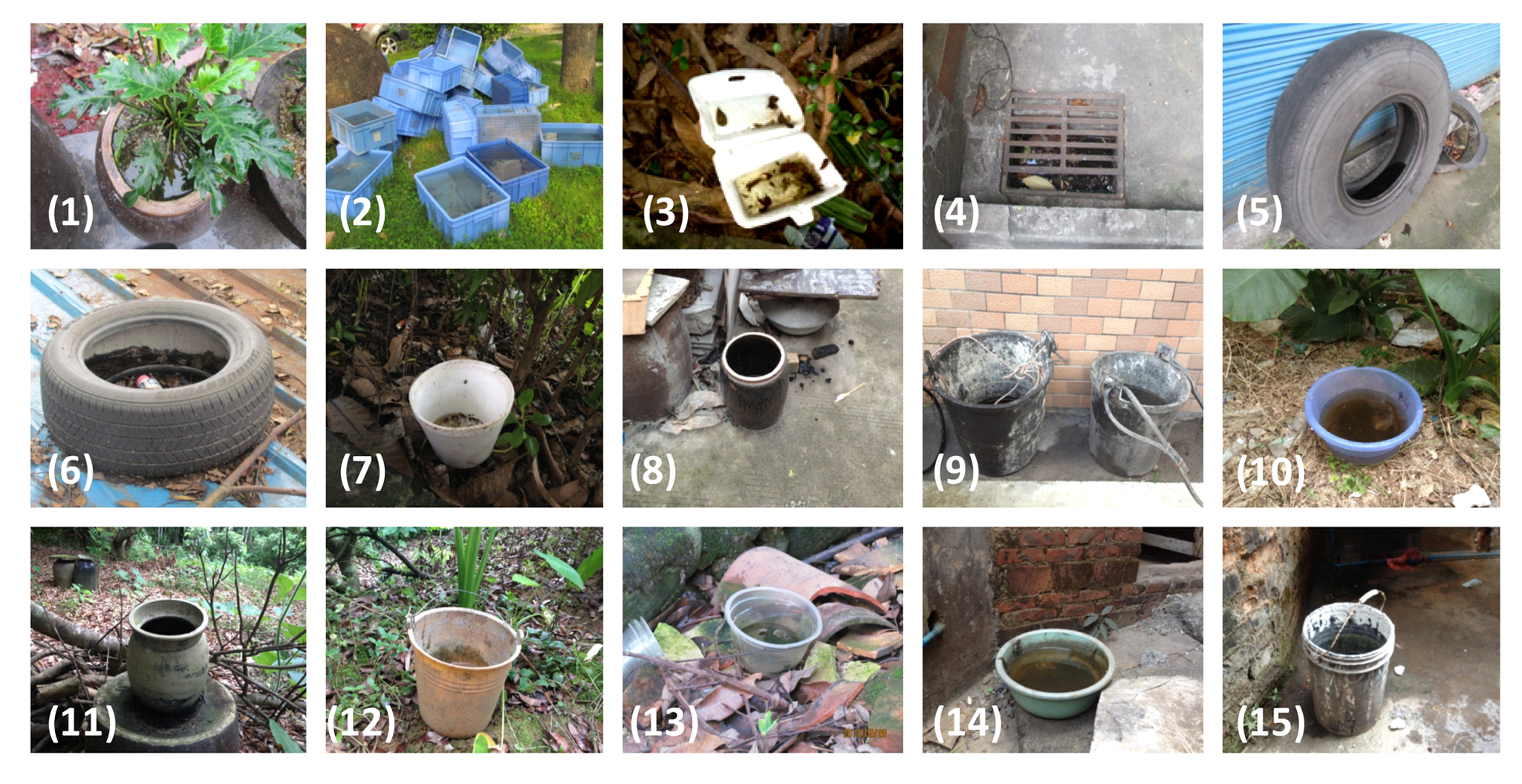

Supplement: Figure S3 — The most abundant Aedes albopictus breeding habitats in the three study sites. Urban area: (1) aquatic plant, (2) plastic bucket, (3) disposable food tin, (4) gutter, (5) tire; suburban area: (6) tire, (7) disposal food tin, (8) clay pottery, (9) plastic bucket, (10) plastic basin; rural area: (11) clay pottery, (12) plastic bucket, (13) disposal food tin, (14) plastic basin, (15) building tool. (TIF) [file pntd.0003301.s003.tif]
