# Supplementary material for: Urbanization Increases Aedes albopictus Larval Habitats and Accelerates Mosquito Development and Survivorship
Source: PLoS Negl Trop Dis. 2014 Nov 13;8(11):e3301. doi: 10.1371/journal.pntd.0003301 (PMC4230920; doi:10.1371/journal.pntd.0003301)
Supplement: Table S1 — Development of Ae. albopictus larvae. Note: Values are the mean ± standard deviation. Values in the same column within the same experimental group connected with the same letter indicate a significant difference at the 5% level. (DOCX) [file pntd.0003301.s004.docx]

**Table S1. Development of *Ae. albopictus* larvae**

| Group | Area | | Average water temperature (℃) | | Mean development time of males (days) | | Mean development time of females (days) | | Adult emergence rate (%) | |  |
| --- | --- | --- | --- | --- | --- | --- | --- | --- | --- | --- | --- |
| Natural habitat | Urban | | 25.77 ± 2.68 a | | 21.39 ± 5.16 a | | 24.15 ± 5.81 a | | 51.50 ± 19.01 a | |  |
|  | Suburban | | 20.88 ± 3.36 b | | 28.33 ± 6.79 b | | 32.82 ± 6.87 b | | 19.25 ± 16.19 b | |  |
|  | Rural | | 20.47 ± 4.10 b | | 31.69 ± 9.01 b | | 33.99 ± 8.87 b | | 13.89 ± 10.29 b | |  |
| Food supplement | | Urban | | 25.77 ± 2.68 a | | 9.57 ± 0.36 a | | 10.36 ± 0.30 a | | 98.00 ± 3.50 a | |
|  | Suburban | | 20.88 ± 3.36 b | | 12.50 ± 0.54 b | | 14.05 ± 3.40 b | | 89.29 ± 10.97 ab | |  |
|  | Rural | | 20.47 ± 4.10 b | | 13.61 ± 2.02 b | | 13.99 ± 1.80 b | | 82.22 ± 9.72 b | |  |

Note: Values are means ± standard deviations. Values in the same column within the same experimental group connected with the same letter indicate no significant difference at level of 5%.
