# Supplementary material for: Urbanization Increases Aedes albopictus Larval Habitats and Accelerates Mosquito Development and Survivorship
Source: PLoS Negl Trop Dis. 2014 Nov 13;8(11):e3301. doi: 10.1371/journal.pntd.0003301 (PMC4230920; doi:10.1371/journal.pntd.0003301)
Supplement: Table S2 — Life table analysis of Ae. albopictus adults. Note: Values are the mean ± standard deviation. Values in the same column connected with the same letter indicate a significant difference at the 5% level within the same experimental group. (DOCX) [file pntd.0003301.s005.docx]

**Table S2. Life table analysis of adult *Ae. albopictus****

| Season | Area | Mean relative | Mean temperature | Median survival time | Median survival time | Mean daily survival | Mean daily survival | |
| --- | --- | --- | --- | --- | --- | --- | --- | --- |
|  |  | humidity (% ) | (℃) | of male (days) | of female (days) | rate of males | rate of females | |
| August - September | Urban | 82.11 ± 11.13 a | 29.35 ± 1.71 a | 14.00 ± 1.05 a | 21.00 ± 1.07 a | 0.87 ± 0.02 a | 0.90 ± 0.02 a |  |
|  | Suburban | 75.91 ± 7.58 b | 29.22 ± 0.94 a | 16.00 ± 0.91 b | 17.00 ± 1.33 b | 0.87 ± 0.02 a | 0.89 ± 0.03 a |  |
|  | Rural | 87.52 ± 9.42 c | 28.05 ± 1.84 b | 13.00 ± 4.12 c | 13.00 ± 0.09 b | 0.88 ± 0.04 a | 0.90 ± 0.04 a |  |
| October - November | Urban | 68.47 ± 13.61 a | 24.79 ± 2.59 a | 18.00 ± 0.07 a | 26.00 ± 1.04 a | 0.89 ± 0.01 a | 0.92 ± 0.01 a |  |
|  | Suburban | 70.52 ± 10.66 a | 22.78 ± 3.85 b | 9.00 ± 0.26 b | 20.00 ± 0.70 a | 0.83 ± 0.04 b | 0.93 ± 0.04 a |  |
|  | Rural | 73.97 ± 12.69 a | 21.86 ± 2.37 b | 11.00 ± 0.19 c | 13.00 ± 0.24 b | 0.81 ± 0.02 b | 0.89 ± 0.03 b |  |

*Note: Values are means ± standard deviations. Values in the same column connected with the same letter indicate no significant difference at level of 5% within the same experimental group.
